# Nitrous oxide emission from wetland soil following single and seasonal split application of cattle manure to field tomato (*Lycopersicon esculentum*, Mill *var. Heinz*) and rape (*Brassica napus*, L. *var. Giant*) crops

**DOI:** 10.1186/s40064-016-1973-3

**Published:** 2016-04-08

**Authors:** Johnson Masaka, Justice Nyamangara, Menas Wuta

**Affiliations:** Department of Land and Water Resources Management, Faculty of Natural Resources Management and Agriculture, Midlands State University, Private Bag 9055, Gweru, Zimbabwe; Department of Environmental Science and Technology, School of Agricultural Sciences and Technology, Chinhoyi University of Technology, P.O. Box 7724, Chinhoyi, Zimbabwe; Department of Soil Science and Agricultural Engineering, Faculty of Agriculture, University of Zimbabwe, Mount Pleasant, P.O. Box MP 167, Harare, Zimbabwe

**Keywords:** Nitrous oxide, Emission, Manure, Wetland, Vegetable

## Abstract

An understanding of the contribution of manure applications to global atmospheric N_2_O loading is needed to evaluate agriculture’s contribution to the global warming process. Two field experiments were carried out at Dufuya wetland (19°17′S; 29°21′E, 1260 m above sea level) to determine the effects of single and split manure applications on emissions of N_2_O from soil during the growing seasons of two rape and two tomato crops. Two field experiments were established. In the first experiment the manure was applied in three levels of 0, 15, and 30 Mg ha^−1^ as a single application just before planting of the first tomato crop. In the second experiment the 15 and 30 Mg ha^−1^ manure application rates were divided into four split applications of 3.75 and 7.5 Mg ha^−1^ respectively, for each of the four cropping events. Single applications of 15 and 30 Mg ha^−1^ manure once in four cropping events had higher emissions of N_2_O than those recorded on plots that received split applications of 3.75 and 7.5 Mg ha^−1^ manure at least up to the second test crop. Thereafter N_2_O emissions on plots subjected to split applications of manure were higher or equal to those recorded in plots that received single basal applications of 30 Mg ha^−1^ applied a week before planting the first crop. Seasonal split applications of manure to wetland vegetable crops can reduce emissions of N_2_O at least up to the second seasonal split application.

## Background

In sub-tropical regions of Africa, manures play an important role in soil fertility management through their short-term effects on nutrient supply and long-term contribution to the soil organic matter. The increasing prices of inorganic fertilizers coupled with growing concerns for sustaining soil productivity has led to renewed interest in the use of cattle manures as fertility-restorer inputs (Mutsamba et al. 2012).

Water is one of the most critical factors that limit smallholder crop production in the semi-arid areas of Zimbabwe. About 74 % of the smallholder areas of the country are located in the southern, western and central in Agro-ecological Regions III, IV and V, where rainfall is generally low and erratic (300–800 mm year^−1^) for reliable dry land cropping by smallholder farmers (Mugandani et al. 2012). The assured availability of water in wetlands which can be extracted without large capital intensive measures has enticed smallholder farmers to intensively utilize wetlands under cropping (Owen et al. 1995). The aerobically composted smallholder cattle manure remains the dominant fertilizer for use by the wetland farmers (Owen et al. 1995).

The addition of cattle manure to wetland soil increases the amount of readily decomposable organic matter associated with high soil microbial activity (Markewich et al. 2010). This enhances the potential for denitrification (Lin et al. 2011) and increased emissions of nitrous oxide (N_2_O) gas through a general stimulation of microbial respiration, causing rapid oxygen consumption and consequently an increase of anaerobic conditions (Yates et al. 2006; Jassal et al. 2011). Flooded soils in wetlands have aerobic and anaerobic zones, allowing both nitrification and denitrification to take place simultaneously (Johnson et al. 2005, Berdad-Haughn et al. 2006). Since the first process produces the substrate for the second, N losses can be very high when the two processes are associated (Snyder et al. 2009). As much as 60–70 % of applied N may be lost as N_2_O (Conrad et al. 1983; Markewich et al. 2010; Kamaa et al. 2011).

Nitrous oxide is a greenhouse and ozone-depleting gas (Mosier and Kroetze 1999; IPCC 2001; Vasileiadou et al. 2011; Mapanda et al. 2012) whose atmospheric concentration is currently >310 nL L^−1^ and increasing at a rate of approximately 0.4 % per annum (Mosier and Kroetze 1999). It is estimated to account for some 6 % of the greenhouse warming (Ma et al. 2007). Nitrous oxide has a global warming potential of 270–320 times compared to carbon dioxide (Snyder et al. 2009; Smith 2012). Nitrous oxide gas can last 150 years in the atmosphere (Munoz et al. 2010; Saggar 2010). The major sink for N_2_O is the stratospheric reaction with atomic oxygen to NO, which induces the destruction of stratospheric ozone. In addition, gaseous losses of manure N as N_2_O reduce the amount of N available to the crop and, therefore, its economic value as fertilizer (Lesschen et al. 2011). Several workers have reported that N_2_O is produced following the breakdown of N compounds in applied manures (Wrage et al. 2004; Wang et al. 2012; Smith 2012) in soil.

Research during the past several decades has improved our understanding of how N_2_O is produced in agricultural systems, the factors that control its production, source/sink relationships, and gas movement processes. However, despite extensive knowledge of the processes involved, researchers are only beginning to be able to predict the fate of a unit of N that is applied or deposited on a specific agricultural field (Mosier et al. 2003). Existing data on emissions of N_2_O is extracted from research generated in western Europe, north America and south-east Asia (Kroetze et al. 2003) despite the fact that the tropics and subtropics contribute greatly to the emissions (Billy et al. 2010), particularly since 51 % of world soils are in these climate zones (Mosier et al. 2003). The incorporation of data on N_2_O emissions from African tropical and sub-tropical regions in the near future will lead to realistic and more appropriate emission factors being used by the IPCC (Kroetze et al. 2003). An understanding of the contribution of manure applications to global atmospheric N_2_O loading is needed to evaluate agriculture’s contribution to the global warming process (Mapanda et al. 2012). We report in this paper on two field experiments conducted over a period of two seasons in 2007 and 2008. The objective of this study was to quantify the effects of single and seasonal split applications of aerobically decomposed cattle manure on N_2_O fluxes from a wetland field during the growing seasons of rape and tomato crops under sub-tropical conditions in Zimbabwe. In this study it was hypothesized that the concentration of mineralized N in wetland soil, N_2_O emissions, N uptake and above ground dry matter yield of tomato and rape crops increase with increasing rates of application of *aerobically composted* cattle manure. It was also hypothesized that seasonal split applications of cattle manure in small doses reduces N_2_O fluxes in soil under rape (*Brassica napus*, L. *var. Giant*) and tomato (*Lycopersicon esculentum*, Mill *var. Heinz*).

## Methods

### Study site description

**Fig. 1:**
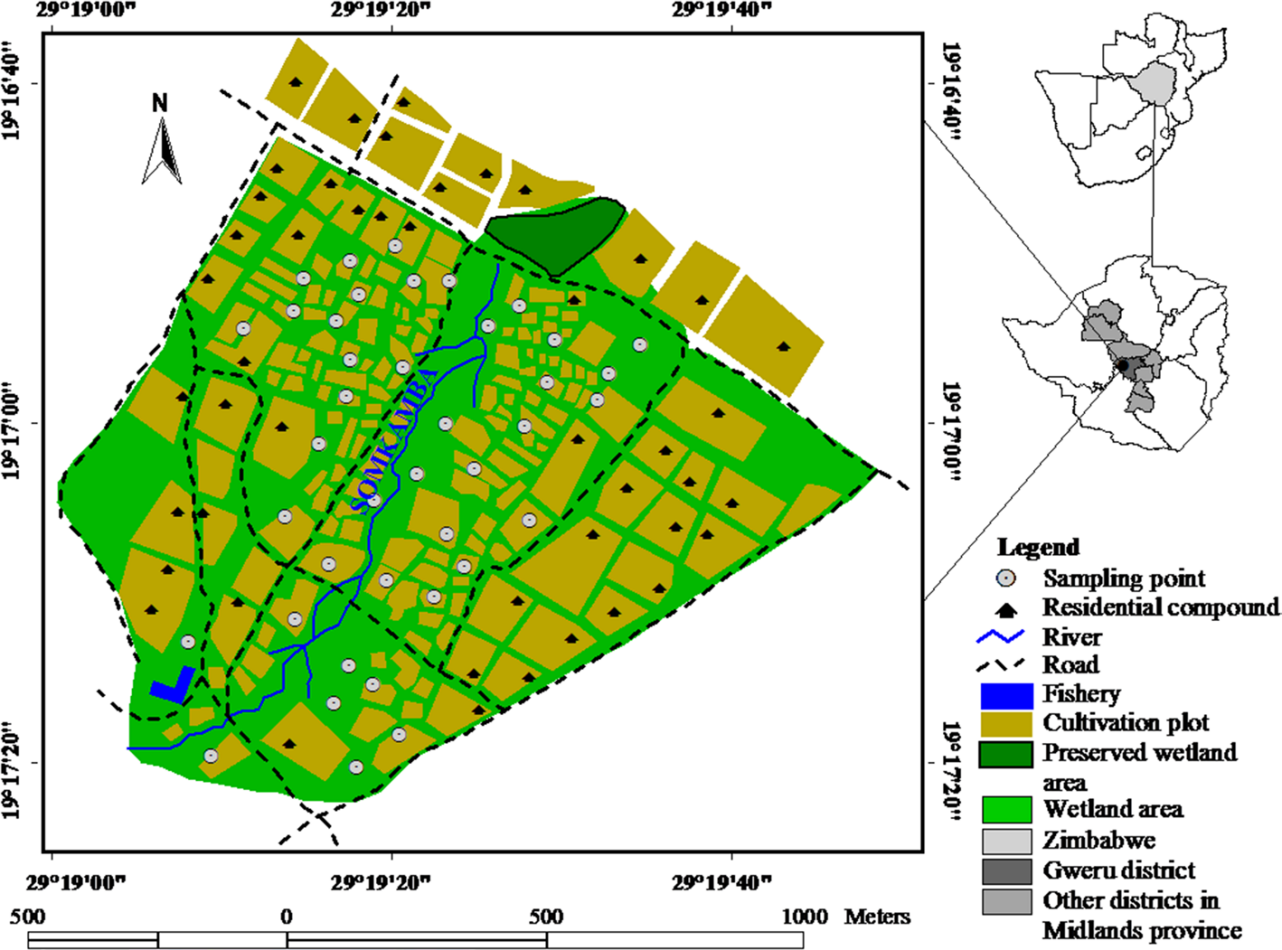
Study site location of Dufuya wetland in Zimbabwe

The study was conducted between 2007 and 2009 in a typical wetland garden at Dufuya (19°17′S; 29°21′E, 1260 m above sea level) wetlands in Chief Sogwala area of Lower Gweru Communal Lands, about 42 km west of the city of Gweru, Zimbabwe (Fig. 1).

The field experimental site is in Agro-ecological Region III, which receives total rainfall ranging from 650 to 800 mm per annum (average 725 mm) and mean annual temperature is 21 °C with insignificant frost occurrence in the months of June and July (Mugandani et al. 2012). Rainfall occurs during a single rainy season extending from November to April. The experimental soil is a deeply weathered course textured loamy sand topsoil over sandy loam subsoil derived from granite and classified as Udic Kandiustalf (USDA) and Gleyic Luvisol (FAO) (FAO 1988; Nyamapfene; 1991 Soil Survey Staff 1992). The soil is perennially moist in part of the profile and smallholder farmers have established vegetable gardens along the wetland. Surface runoff and seepage of groundwater from catchment areas over an impermeable substratum towards lower lying areas, together with incident precipitation contribute largely to the water budget of the wetland. Vegetable production is all year round. The site had been under alternate rape, tomato, and maize crops for several years. Rape is cultivated as a leaf vegetable in Zimbabwe (De Lannoy 2001).

### Characterization of experimental soil

Initial soil characterization was done by collecting twenty soil samples from randomly selected points of the field experimental site at a depth of 0–20 cm using a soil auger. Organic C in soil was determined using the Walkely and Black method (Nelson and Sommers 1996). Soil texture was determined by the Bouyocous hydrometer method (Bouyoucos 1965). Soil bulk density was determined by the core method (Black and Hartge 1986). The soil cores were oven-dried at 105 °C (to constant weight) for determination of mean gravimetric water content. Taking particle density (Pd) of soil to be 2.65 g cm^−3^ total porosity was calculated and recorded. Total N in soil was measured by the Kjeldahl method described by Bremner (1996). Results of the analyses are shown in Table 1.

**Table 1:** Chemical and physical properties of the experimental soil

Soil depth (cm)	Soil pH (H_2_O)	Org-C (%)	^1^N (mg kg^−1^)	Sand (%)	Clay (%)	Silt (%)	Total porosity (cm^3^ cm^−3^)	Bulk density (g cm^−3^)	Saturation gravimetric water (g g^−1^)
0–20	5.5	0.4	24	85	10	5	0.46	1.28	0.51
20–60	5.8	0.2	20	80	15	5	0.43	1.34	0.67
60–100	5.7	0.2	20	78	17	5	0.41	1.39	0.69

### Land preparation and crop management

The land was prepared by digging using hand hoes to a depth of 30 cm and then leveling using a rake. Plots raised to a height of 15 cm, which measured 5 × 1.5 m, were then carefully marked out. The distance between the plots was 60 cm. Small 20 cm high ridges were established around each plot to avoid cross-contamination by surface run-off. Tomato and rape crops were used as test crops in the study. The cropping sequence in the field experiment was: September–December 2007 first tomato, January–March 2008 first rape, April–July 2008 second tomato and September–November 2008 second rape crops. Spacing between rows was 30 and 15 cm within the rows for the rape crop. For the tomato crop the plant spacing was 90 cm between rows and 80 cm within rows.

### Experimental manure

The smallholder farmers at Dufuya wetlands practice intensive tomato and rape production in small gardens under small scale irrigation (Owen et al. 1995). Because of lack of availability and higher cost of chemical fertilizers, the smallholder farmers have resorted to use of cattle manure which are readily available. The aerobically composted cattle manure used in the field plot experiment was collected from a homestead in the surrounding communal area. High rates of manure applications are used in order to avoid yield depression due to nutrient deficiency (Owen et al. 1995; De Lannoy 2001). Usually, 15 Mg ha^−1^ of cattle manure is applied by wetland farmers with limited number of cattle (<6). On average, 30 Mg cattle manure ha^−1^ is applied by wetland farmers with larger cattle herds (>6). Smallholder farmers in the wetland may apply these doses once in four cropping events because of the limited annual accumulations of manure in cattle holding pens. In some cases, smaller doses of cattle manure (3–8 Mg ha^−1^) in every cropping event are applied by farmers with a smaller herd of cattle. These manure application rates and seasonal split applications were used as treatments in the field experiments in order to capture the common farmer practice and test their effects on loss of N through N_2_O emissions.

Ten randomly selected samples were collected from a pile of manure and thoroughly mixed in a plastic bucket. Three replicate composite samples were taken for laboratory analysis. The samples were air-dried, passed through a 2 mm sieve, and analyzed for organic C (Nelson and Sommers 1982), total N using the Kjeidahl procedure (Bremner and Mulvaney 1982), soil, and ash content. Soil and ash contents were determined by ashing manure in a muffle furnace (450 °C) for 16 h. The ash was dissolved in concentrated HCl acid and separated from mineral soil by filtering. The soil was oven dried and weighed. The selected chemical properties of the experimental manure are shown in Table 2.

**Table 2:** Selected chemical properties of the smallholder cattle manure

Organic C (%)	Total N (%)	C:N ratio	Soil + ash content (%)	Soil and ash-free basis (%)	
				Organic C	Total N
22.82	1.36	16.8:1	77.18	61.3	6.4

### Experimental design and treatments

Two experiments were used to determine the effect of manure application rates and seasonal split applications on N_2_O emission with three treatments for each experiment:

Experiment 1:

1. Control (unamended);
2. 15 Mg manure ha^−1^ (applied once in four successive cropping events);
3. 30 Mg manure N ha^−1^ (applied once in four successive cropping events).

Experiment 2:

1. Control (unamended);
2. 15 Mg manure ha^−1^ (in four seasonal split applications);
3. 30 Mg manure ha^−1^ (in four seasonal split applications).

A randomized complete block design with four replications was employed. The blocking factor was the slope gradient. In Experiment 1, the 15 and 30 Mg manure ha^−1^ were applied once in four cropping events in the respective plots by broadcasting on the surface and then incorporating into the soil just before transplanting the first tomato crop. In Experiment 2, the 15 and 30 Mg ha^−1^ manure rates, applications were divided into four split seasonal applications over the study period in which two tomato and two rape crops were planted. For the 15 Mg ha^−1^ cattle manure treatments, the first application of 3.75 Mg ha^−1^ was done by evenly applying manures in planting rows on the raised plots and then incorporating it a few days before planting the first tomato crop. The balance of three applications of 3.75 Mg ha^−1^ was applied to each of the remaining three crops in the study by applying into the planting furrows and covering with soil before planting each crop. The same seasonal split application procedure was repeated for the 30 Mg ha^−1^ manure treatments, which was divided into four applications of 7.5 Mg ha^−1^ for each of the four crops.

A basal application rate of 1000 kg ha^−1^ compound S (5 % N, 7.9 % P, 16.6 % K, and 8 % S) was used in all treatments before planting each crop to capture common fertilizer application practice at Dufuya wetland.

### Weather conditions

**Fig. 2:**
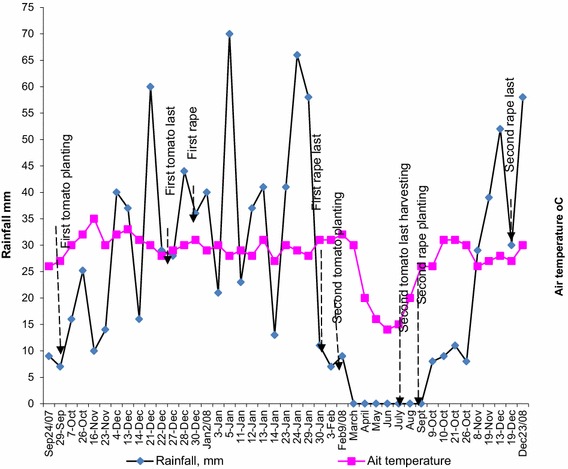
Daily rainfall, air temperature at the study site

Rainfall data were collected daily at 10.00 h from a rain gauge at the study site. Maximum and minimum daily temperatures at the study site were gap-filled using the department of Agricultural Technical and Extension Services (AGRITEX) meteorological data at Sogwala (19°17′S; 29°21′E) rural service centre located 2 km west of the study site. The meteorological station records daily weather data (Fig. 2).

### Static chamber set-up and N_2_O flux measurement

Nitrous oxide emissions from soil were trapped using static chamber method described by Holland et al. (1999) and Meyer et al. (2001). There were seven gas sampling campaigns at 14 day interval for the tomato crop. Six gas sampling events were performed at 14 day interval for the rape crop. Gas sampling was done at time 0 min to obtain the start values of atmospheric concentration of N_2_O in the static chamber head space and after 30 and 60 min (Mathias et al. 1980; Kaiser et al. 1996). The gas samples were analyzed for N_2_O concentration by means of a Varian Model 3400 gas chromatograph (Walnut Creek, CA, USA) as described by Mosier and Mack (1980) and Galle et al. (2003). Nitrous oxide fluxes (Fn) were calculated using the Hutchinson and Livingston (1993) model:

1 $${\text{Fn}} = \frac{\delta Cn}{\delta t} . \frac{\text{V}}{A} . \frac{\text{Mn}}{V\,\hbox{mol}}$$

where $\delta Cn/ \delta t$ is the rate of change in N_2_O concentration (µmol mol^−1^ min^−1^), V is the chamber headspace volume (m^3^), Mn is the molecular weight of N_2_O (44 g mol^−1^), A is the surface area (m^2^) and *Vmol* is the volume of 1 mol of gas at 20 °C (0.024 m^3^ mol^−1^). Further conversions were performed to calculate Fn fluxes in g ha^−1^ day^−1^ as follows (Eq. 2):

2 $${\text{Fn}}\,{\text{g}}\,{\text{ha}}^{ - 1} \,{\text{day}}^{ - 1} = {\text{N}}_{2} {\text{O}}\,{\text{g}}\,{\text{h}}^{ - 1} \cdot 24\, {\text{h}} \cdot \frac{A}{10000}$$

Total N lost as N_2_O (N kg ha^−1^) was calculated using Eq. 3:

3 $${\text{N}}\,{\text{kg}}\,{\text{ha}}^{ - 1} = {\text{Fn}}\,{\text{g}}\,{\text{ha}}^{ - 1} \,{\text{day}}^{ - 1} \cdot \frac{{T\,{\text{days}}}}{1000} \cdot \frac{28}{44}$$

where T is the number of days with similar daily N_2_O emissions rates and 28/44 is the conversion ratio for converting N_2_O molar mass to N content.

#### Soil mineral N measurements

At the same time that gas samples were collected, soil samples (n = 4 per plot) were also collected from the plots and analyzed for NH_4_-N and NO_3_-N. The soil samples were collected from a depth of 0 to 20 cm using a soil auger. Both analyses were performed using an Alpkem 3550 Flow Injector Analyzer (01 Analytical, College Station, TX, USA) using colorimetric techniques (Robertson et al. 1999).

#### Dry matter yield

Four randomly selected plants were chosen and labeled in each plot for crop biomass sampling. All rape leaves and tomato fruits that reached horticultural maturity were harvested from the selected plants at every harvesting event and taken to the laboratory. The samples were rinsed; oven dried at 65 °C for 24 h and kept in a dry place. At the end of the growing season, the aboveground biomass of the selected plants was summed up. The composite samples were then ground to pass a 2 mm sieve and analyzed for N concentration semi-micro Kjeldahl procedure (Bremner and Mulvaney 1982). Total uptake of N was determined by multiplying the N concentration with dry matter yield as follows (Eq. 4):

4 $$N\,uptake\,{\text{kg/ha}} = [N] \cdot DM$$

where [N] is content of N in mg g^−1^ dry matter and DM is dry matter yield in T ha^−1^.

Mineralized N concentrations in soil were monitored at 2-week internals for each treatment and estimated over 98 and 84 days for tomato and rape crops respectively.

### Statistical analysis

Treatment effects on measured variables in each experiment were analyzed using one way ANOVA (GenStat Discovery Edition 3 2003; GenStat VSNI 2011). Differences between treatment means were judged significant at p ≤ 0.05 as determined by Fisher’s protected least significant difference (LSD) test. Flux data were log-transformed to normalize the distributions before the statistical analysis. Mean separation was performed using the LSD since there were not >3 treatments in each set of experiment. Statistical significance of the differences between measured variables in plots subjected to single and seasonal split manure applications was established by performing t test for unpaired samples using the GenStat package. The Pearson coefficients of determination between measured variables and their r^2^ values were computed using Microsoft Excel. Significance of correlations between selected variables was established using a linear model GenStat analysis of correlation at 5 % level.

## Results

### NH_4_-N concentrations in soil following single and split application of manure

**Fig. 3:**
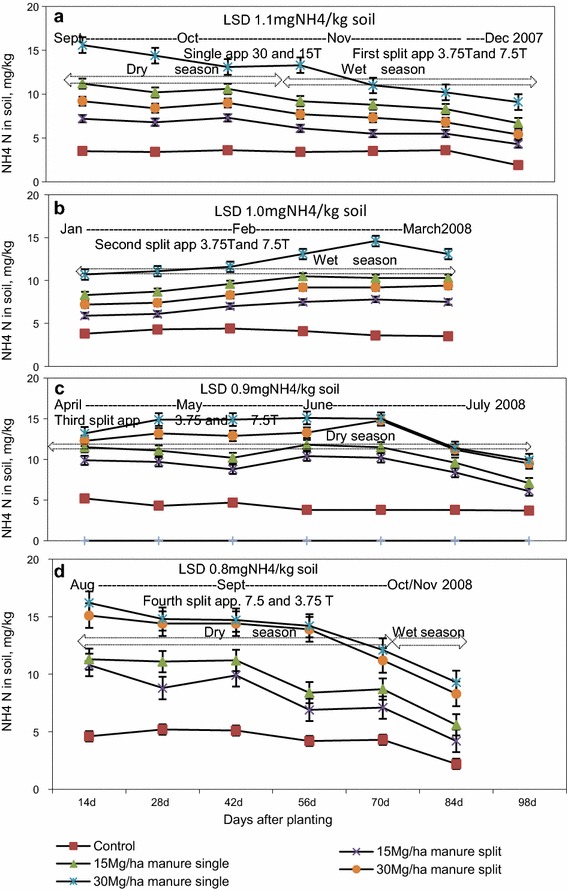
NH_4_-N concentration in wetland soil following single and split application of manure. *app* application. **a** First tomato crop, **b** first rape crop, **c** second tomato crop and **d** second rape crop

The concentration of NH_4_-N in soil subjected to single application was significantly (p < 0.05) higher than that in soil subjected to seasonal split applications during the growing period of the first tomato and rape crops (Fig. 3a, b). However, only rates of manure applications had a significant (p < 0.05) effect on the differences in the concentrations of NH_4_-N during the growing seasons of the second tomato and rape crops. The effect of single and split application of cattle manure on NH_4_-N concentration was not significant (p > 0.05) during the growing period of the second tomato and rape crops (Fig. 3c, d). Except for the first rape crop, NH_4_-N concentrations decreased steadily towards the end of the growing period for each crop.

Single applications of 15 Mg of manure increased the concentration of NH_4_-N in soil by 2.3 (30 %) and 2.0 mg kg^−1^ soil (27 %) above those recorded on plots amended with the first and second split application of 3.75 Mg manure ha^−1^ for the first tomato and rape crops, respectively. Single applications of 30 Mg manure ha^−1^ increased NH_4_-N concentration in soil by 2.9 (29 %) and 2.3 mg kg^−1^ soil (21 %) above those recorded in plots subjected to the first and second split 7.5 Mg ha^−1^ manure applications for the first tomato and rape crops.

### NO_3_-N concentration in soil following single and split application of manure

**Fig. 4:**
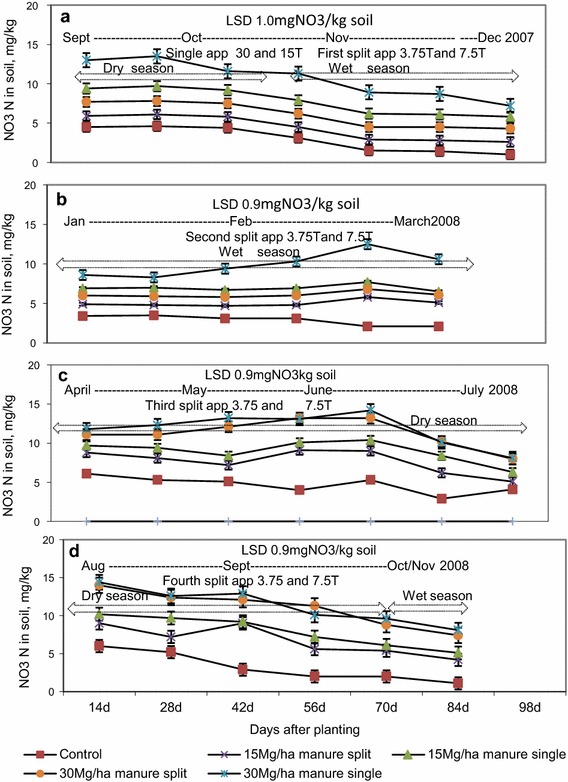
NO_3_-N concentration in wetland soil follow ing single and split application of manure. *app* application. **a** First tomato crop, **b** first rape crop, **c** second tomato crop and **d** second rape crop

Trends for NO_3_-N and NH_4_-N concentrations in soil were comparatively similar during the growing period of test crops. Effects of single and split applications of manure on NO_3_-N were significant (p < 0.05) only up to the second split application while their effects became insignificant (p > 0.05) in the third and fourth split applications (Fig. 4).

Generally, there were significant temporal variations in the concentrations of NO_3_-N in soil from planting up to the cessation of the growing period of each vegetable crop. Application of 15 Mg ha^−1^ manure once in four cropping events significantly increased (p < 0.05) the content of NO_3_-N in wetland soil by 2.4 (40 %) and 1.6 mg kg^−1^ soil (27 %) above those recorded on plots amended with the first and second split application of 3.75 Mg manure ha^−1^ for the first tomato and rape crops.

Single applications of 30 Mg manure ha^−1^ significantly (p < 0.05) increased NO_3_-N concentrations in soil by 2.4 (27 %) and 1.8 mg kg^−1^ soil (21 %) above those recorded in plots amended with first and second split 7.5 Mg ha^−1^ manure for the first tomato and rape crops, respectively. When a single application of 15 and 30 Mg ha^−1^ manure were used instead of 3.75 and 7.5 Mg ha^−1^ applied as a third split application mean NO_3_-N concentration differences between the two treatments approached similar levels and were insignificant in the second tomato and rape crops (third and fourth split applications). The mean differences in the concentrations of NO_3_-N in wetland soil between plots amended with single applications at the beginning of the experiment and split applications before planting the successive test crops progressively became narrower towards the end of the experiment.

### Nitrous oxide fluxes from soil following single and split application of manure

**Fig. 5:**
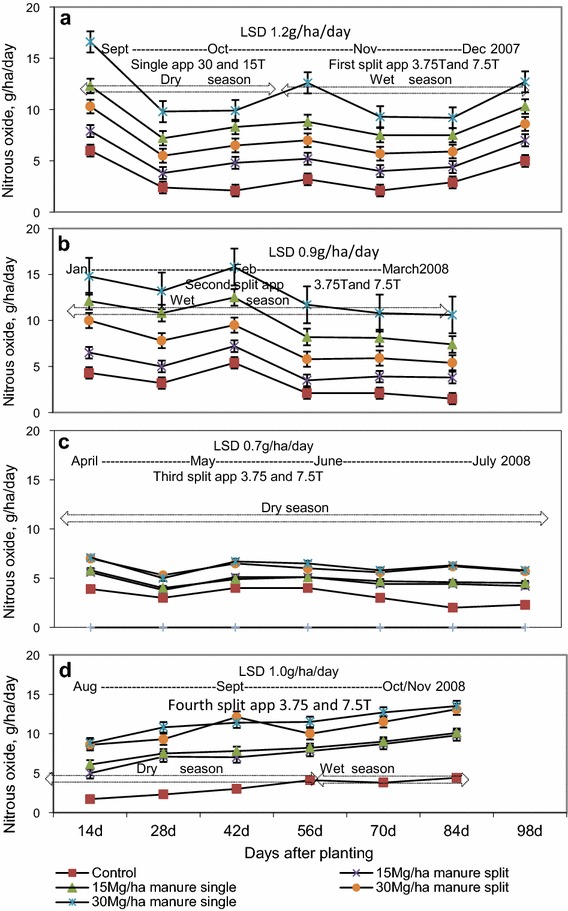
N_2_O fluxes from wetland soil following single and split application of manure. *app* application. **a** First tomato crop, **b** first rape crop, **c** second tomato crop and **d** second rape crop

Results show that the rate of cattle manure applications exerted significant differences (p < 0.05) in N_2_O fluxes following single and seasonal split manure applications throughout the study period (Fig. 5). Nevertheless, split application of manure exerted significant (p < 0.05) effect on N_2_O emissions within the growing periods of the first tomato and rape crops only (Fig. 4a, b) when compared with the control.

Considerably higher N_2_O emissions were observed in the first gas samples collected from vegetable plots amended with single applications of 30 Mg ha^−1^ manure, which was applied a week before planting the first tomato crop. In single manure applications, elevated N_2_O fluxes persisted throughout the 98 and 84-day period for tomato and rape crops respectively. In split applications of manure, N_2_O fluxes remained constant or gradually decreased despite additions of cattle manure before each planting event.

Single applications of 15 Mg manure ha^−1^ increased N_2_O fluxes by 1.8 (36 %) and 2.7 g ha^−1^ day^−1^ (43 %) above those recorded from plots subjected to the first and second split application of 3.75 Mg manure ha^−1^ applied a week before planting the first crop for the tomato and rape crops, respectively. The same practice at 30 Mg manure ha^−1^ application levels increased N_2_O fluxes on wetland soil by 2.5 (38 %) and 3.1 g ha^−1^ day^−1^ (34 %).

### Soil factors–N_2_O emission relationships

**Fig. 6:**
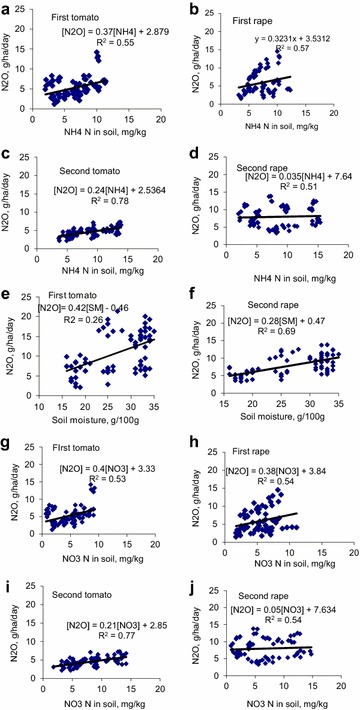
Regression analyses showing relationships between mineral N, N_2_O and wetland soil moisture after split application of manure

**Fig. 7:**
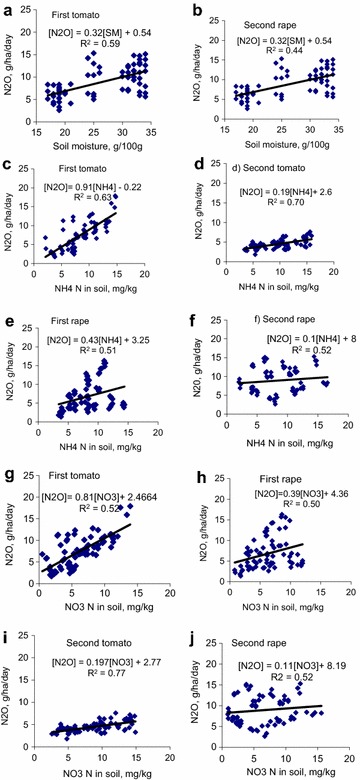
Regression analyses showing relationships between mineral N, N_2_O and wetland soil moisture after a single application of manure

The concentrations of NH_4_-N and NO_3_-N in soil are important predictors of N_2_O fluxes in soil (Figs. 6, 7). Regression analysis between measured variables after split and single application of cattle manure are shown in Figs. 6 and 7. Results show significant correlations (p < 0.05) between NO_3_-N; NH_4_-N; soil moisture and emissions of N_2_O. Coefficients of regression in the correlations between soil moisture and N_2_O emissions varied between 0.26 and 0.69 (Figs. 6e, f, 7a, b). The coefficients of regression (r^2^) values for the positive linearity in the relationships between NH_4_-N concentrations in soil and N_2_O emissions ranged from 0.42 to 0.78 after split and single manure application. The coefficients of determination in the relationships between NO_3_-N in soil and N_2_O fluxes on soil varied between 0.47 and 0.77. The r^2^ values the relationships between NH_4_-N, NO_3_-N in soil and emissions of N_2_O were comparatively similar.

### Aboveground dry matter yield and N uptake following split and single application of manure

Dry matter yield and N uptake following seasonal split and single application of manure are shown in Tables 3 and 4. The effects of single and split applications of manure on N uptake were significant (p < 0.05) for all vegetable crops. However, the differences in dry matter yield between plots subjected to single applications and those amended with the first split applications were larger than those recorded between single manure applied plots and the plots amended with the fourth split application of manure.

**Table 3:** Aboveground dry matter yield and N uptake after split application of manure

Trts	First tomato			First rape			Second tomato			Second rape		
	DM yield T ha^−1^	mg N g^−1^ DM	N uptake (kg ha^−1^)	DM yield (T ha^−1^)	mg N g^−1^ DM	N uptake (kg ha^−1^)	DM yield (T ha^−1^)	mg N g^−1^ DM	N uptake (kg ha^−1^)	DM yield (T ha^−1^)	mg N g^−1^ DM	N uptake (kg ha^−1^)
T1	3	9.8	29.3	9.9	1.6	14.9	3.1	12.9	40.3	10.5	2.9	29.9
T2	3.3	10.4	33.6	11.2	2.9	32.8	4	15.5	62.0	13.3	6.2	82.8
T3	3.6	14.1	50.6	12.7	7.7	98.0	5.8	17	100.2	15	9.9	148.5
Fpr	*	*	*	*	*	*	*	*	*	*	*	*
LSD (5 %)	0.1	3	3.9	0.2	0.2	1.4	0.1	2.3	2.7	0.2	0.5	5.6
CV %	0.9	15.3	5.9	1.2	2.7	1.6	1.8	10.4	2.3	1	4.2	4.5

T1—control, T2—15 Mg high N manure ha^−1^ split into four 3.75 Mg ha^−1^ per crop, T3—30 Mg high N manure ha^−1^ split into four 7.5 Mg ha^−1^ per crop, DM—dry matter yield, mg N g^−1^ DM—milligrams of N per gram dry matter

* p > 0.05

**Table 4:** Dry matter yield and N uptake by aboveground plant biomass following single application of manure

Trts	First tomato (2007–2008)			First rape (2008–2009)			Second tomato (2008–2009)			Second rape (2008–2009)		
	DM yield (T ha^−1^)	mg N g^−1^ DM	N uptake (kg ha^−1^)	DM yield (T ha^−1^)	mg N g^−1^ DM	N uptake (kg ha^−1^)	DM yield (T ha^−1^)	mg N g^−1^ DM	N uptake (kg ha^−1^)	DM yield (T ha^−1^)	mg N g^−1^ DM	N uptake (kg ha^−1^)
T1	3.0	7.0	20.9	10.5	1.5	9.1	3.1	9.1	28.2	10.1	2.2	32.3
T2	5.5	11.7	79.6	12.0	4.6	57.5	7.0	7.1	55.7	11.0	3.2	59.2
T3	8.5	17.2	146.2	16.5	8.5	136.2	9.5	16.1	138.2	16.5	5.8	121.8
Fpr	*	*	*	*	*	*	*	*	*	*	*	*
LSD (5 %)	0.1	0.8	0.7	1.4	1.3	0.5	0.5	1.6	1.2	1.0	1.2	0.6
CV %	1.0	4.1	5.3	6.2	15.4	5.4	4.2	6.6	7.4	3.8	12.4	6.8

Trts—treatments, T1—control, T2—15 Mg manure ha^−1^ applied once in four cropping events, 30 Mg high N manure ha^−1^ applied once in four cropping events, DM—dry matter yield, mg N g^−1^ DM—milligrams of N per gram dry matter

N uptake was lowest in the control plots and highest in plots that received 30 Mg manure as a single application. Plots amended with split applied manure recorded substantial reductions in N uptake when compared with those recorded on plots amended with single manure applications. When 15 and 30 Mg manure ha^−1^ were applied once, N uptake increased by 48.3 kg ha^−1^ or 59 % and 102 kg N ha^−1^ or 67 % in excess of those recorded in plots amended with the first split applications of 3.75 and 7.5 Mg manure ha^−1^, respectively.

The second tomato crop experienced increase of N uptake of 63.4 kg ha^−1^ or 51 % and 76.0 kg ha^−1^ or 43 % in plots subjected to single applications of 15 and 30 Mg manure ha^−1^ in comparison with those observed in plots amended with the third split applications of 3.75 and 7.5 Mg manure ha^−1^ respectively.

While N uptake responses to single applications of 15 and 30 Mg high N manure ha^−1^ were 37–67 % above those in plots subjected to split applications of 3.75 and 7.5 Mg manure ha^−1^ for the three previous crops, the same soil fertilization practice could increase N uptake by only 3.4 kg ha^−1^ or 4 % and 10.5 kg ha^−1^ or 7 % respectively for the last crop in the study.

Single applications of high N manure at 15 Mg ha^−1^ stimulated an increase of 51, 11, 42, and 19 % in dry matter yield in excess of those recorded on plots subjected to the first, second, third and fourth split applications of high N manure. The application of 30 Mg manure ha^−1^ once during the study period caused an increase in dry matter yield of 58, 23, 23, and 9 % during the first, second, third and fourth split application of 7.5 Mg ha^−1^ manure.

### Total N lost as nitrous oxide

Tables 5 and 6 shows estimated losses of N in N_2_O emission after seasonal split and single applications of manure to rape and tomato crops. Single applications of cattle manure had a significant effect on losses of N_2_O from soil throughout the growing period of tomato and rape crops (Table 4). The effect of split applications of manure on emissions of N_2_O were significant (p < 0.05) during the growing period of the first tomato and rape crops only (Table 3). Thereafter, the rates of application rather than the factors of single and seasonally split manure applications had significant effect on total N lost through N_2_O emissions. Losses of N through N_2_O emission on plots amended with split applications of manure were 23–138 % above the losses recorded on the control plots. Estimated total N lost through N_2_O emissions on plots subjected to single applications of manure were 121 and 134 % above the emissions recorded on control plots. Amongst the manure amended plots, lower N losses of N_2_O emission were recorded in the second tomato, a crop which grew under dry weather conditions of the 2008 April–July winter season.

**Table 5:** Estimated total N lost through nitrous oxide emission following seasonal split application of manure

Trts	First tomato crop					First rape crop				
	Temporal interval (days after planting)	Mean rate of N_2_O emission (g ha^−1^ day^−1^)	Total N emitted for the period (kg ha^−1^)	Total N applied (kg ha^−1^)	% emitted N_2_O of applied N	Temporal interval (days after planting)	Mean rate of N_2_O emission (g ha^−1^ day^−1^)	Total N emitted for the period (kg ha^−1^)	Total N applied (kg ha^−1^)	% emitted N_2_O of applied N
T1	1–21	5.4	0.11	–	–	1–49	6.0	0.30	–	–
	22–49	2.5	0.07	–	–	50–84	3.9	0.13	–	–
	50–63	2.8	0.04	–	–	–	–	–	–	–
	64–98	6.4	0.22	–	–	–	–	–	–	–
Total			0.44	0	0	–	–	0.43	0	0
T2	1–21	5.6	0.12	–	0.24	1–49	9.3	0.45	–	0.88
	22–49	5.7	0.15	–	0.29	50–84	4.6	0.15	–	0.29
	50–63	5.7	0.07	–	0.14	–	–	–	–	–
	64–98	7.2	0.24	–	0.47	–	–	–	–	–
Total	–	–	0.58	51	1.14	–	–	0.60	51	1.18
T3	1–21	7.5	0.15	–	0.15	1–49	12.7	0.62	–	0.61
	22–49	6.8	0.16	–	0.16	50–84	7.2	0.24	–	0.24
	50–63	6.5	0.08	–	0.08	–	–	–	–	–
	64–98	9.4	0.31	–	0.15	–	–	–	–	–
Total	–	–	0.70	102	0.69	–	–	0.86	102	0.84
Fpr	–	–	*	–	–	–	–	*	–	–
LSD	–	–	0.16	–	–	–	–	0.23	–	–
CV	–	–	13.40	–	–	–	–	12.20	–	–

	Second tomato crop					Second rape crop				
T1	1–98	3.3	0.32	–	–	1–35	3.0	0.11	–	–
–	–	–	–	–	–	36–84	4.3	0.21	–	–
Total			0.32	0	–			0.32	0	–
Trt2	1–98	5.1	0.50	–	0.98	1–35	5.2	0.18	–	0.35
–	–	–	–	–	–	36–84	7.3	0.35	–	0.68
Total			0.50	51	0.98	–	–	0.53	51	1.04
T3	1–98	5.9	0.58	–	0.57	1–35	7.7	0.27	–	0.26
–	–	–	–	–	–	36–84	10.2	0.49	–	0.48
Total	–	–	0.58	102	0.57	–	–	0.76	102	0.75
Fpr	–	–	*	–	–	–	–	*	–	–
LSD	–	–	0.14	–	–	–	–	0.24	–	–
CV	–	–	15.70	–	–	–	–	8.80	–	–

Trt—treatments, T1—control, T2—15 Mg high N manure ha^−1^ split into four 3.75 Mg ha^−1^ applications per crop, T3—30 Mg high N manure ha^−1^ split into four 7.5 Mg ha^−1^ applications per crop

* p > 0.05

**Table 6:** Estimated total N lost through N_2_O emission following single application of manure

Trts	First tomato crop					First rape crop				
	Temporal interval (days after planting)	Mean rate of N_2_O emission (g ha^−1^ day^−1^)	Total N emitted for the period (kg ha^−1^)	Total N applied (kg ha^−1^)	% emitted N_2_O of applied N	Temporal interval (days after planting)	Mean rate of N_2_O emission (g ha^−1^ day^−1^)	Total N emitted for the period (kg ha^−1^)	Total N applied (kg ha^−1^)	% emitted N_2_O of applied N
T1	1–21	5.6	0.12	–	–	1–49	6.0	0.30	–	–
	22–49	2.4	0.07	–	–	50–84	3.9	0.13	–	–
	50–63	2.7	0.04	–	–	–	–	–	–	–
	64–98	6.0	0.21	–	–	–	–	–	–	–
Total			0.44	0	0	–	–	0.43	0	0
T2	1–21	8.1	0.17	–	0.08	1–49	10.1	0.50	–	0
	22–49	7.1	0.19	–	0.09	50–84	5.8	0.40	–	0
	50–63	7.0	0.09	–	0.04	–	–	–	–	–
	64–98	9.1	0.31	–	0.15	–	–	–	–	–
Total	–	–	0.76	204	0.37	–	–	0.90	0	0
T3	1–21	13.5	0.28	–	0.07	1–49	14.1	0.70	–	0
	22–49	9.0	0.24	–	0.06	50–84	10.1	0.30	–	0
	50–63	8.0	0.10	–	0.02	–	–	–	–	–
	64–98	10.3	0.35	–	0.09	–	–	–	–	–
Total	–	–	0.97	408	0.24	–	–	1.00	0	0
Fpr	–	–	*	–	–	–	–	*	–	–
LSD	–	–	0.16	–	–	–	–	0.23	–	–
CV	–	–	13.40	–	–	–	–	12.20	–	–

	Second tomato crop					Second rape crop				
Trt1	1–98	3.3	0.32	–	–	1–35	3.0	0.12	–	–
–	–	–	–	–	–	36–84	4.3	0.21	–	–
Total			0.32	0	–			0.33	0	–
T2	1–98	5.2	0.51	**–**	0.6	1–35	5.1	0.22	–	0
–	–	–	–	**–**	–	36–84	7.4	0.43	–	0
Total			0.51	0	0			0.65	0	0
T3	1–98	6.1	0.60	–	0	1–35	7.6	0.27	–	0
–	–	–	–	–	–	36–84	10.0	0.48	–	0
Total	–	–	0.60	0	0	–	–	0.75	0	0
Fpr	–	–	*	–	–	–	–	*	–	–
LSD	–	–	0.14	–	–	–	–	0.24	–	–
CV	–	–	15.70	–	–	–	–	8.80	–	–

Trts—treatments, T1—control, T2—15 Mg high N manure ha^−1^, T3—30 Mg high N manure ha^−1^

* p > 0.05

When 15 Mg ha^−1^ of manure were applied once in the four cropping events N_2_O emission increased by 31 and 38 % above those recorded from plots subjected to the first and second split application of 3.75 Mg manure ha^−1^ applied a week before planting the first tomato and rape crops, respectively. Mean differences in total N lost as N_2_O emission between plots amended with a single basal application of 30 Mg of manure and those amended with the first and second seasonally split application of 7.5 Mg manure ha^−1^ were 39 and 13 % for the first tomato and rape crops respectively. As the study approached the last cropping event, mean differences in the loss of N through N_2_O emissions between plots amended with single basal applications and those that received seasonally split applications became progressively smaller and insignificant.

When 15 and 30 Mg manure ha^−1^ were applied once in four cropping events 0.4 and 0.9 % of applied N was lost as N_2_O, respectively, during the growing period of the first tomato crop. When 15 and 30 Mg of manure were split applied into four applications of 3.75 and 7.5 Mg ha^−1^ to every crop total N losses in N_2_O emission represented 0.9 and 0.9 % (for the rape crop); 0.8 and 0.6 % (for the tomato crop) of applied N. Generally, the proportion of applied N lost as N_2_O was higher in the rape crop than in the tomato crop.

#### Total N lost in N_2_O emission per unit dry matter

Table 7 shows N lost in N_2_O emission per unit of harvested dry matter yield after the single application of cattle manure in four vegetable cropping events. When the application rates of manure were increased from 15 to 30 Mg ha^−1^, the emissions of N_2_O per unit harvested dry matter of rape and tomato significantly decreased (p < 0.05). The estimated loss of N in N_2_O emissions decreased by 0.01–0.03 and 0.01–0.06 kg N-N_2_O per T of harvested dry matter when manure application rates were increased from 15 to 30 Mg ha^−1^, respectively. Nitrous oxide emission losses per unit harvested dry matter of tomato crop were significantly (p < 0.05) higher in the unamended plots than on manure fertilized plots (Table 7). However, losses of N in N_2_O emissions per unit harvested dry matter from the control plots under rape crop were generally lower when compared with the losses from manure fertilized plots.

**Table 7:** Estimated N lost in N_2_O emission per unit dry matter yield after single manure application

Trts	First tomato			First rape			Second tomato			Second rape		
	DM yield (T ha^−1^)	N emitted in N_2_O (kg ha^−1^)	kg N emitted per T DM	DM yield (T ha^−1^)	N emitted in N_2_O (kg ha^−1^)	kg N emitted per T DM	DM yield (T ha^−1^)	N emitted in N_2_O (kg ha^−1^)	kg N emitted per T DM	DM yield (T ha^−1^)	N emitted in N_2_O (kg ha^−1^)	kg N emitted per T DM
T1	3.0	0.44	0.15	10.5	0.43	0.04	3.1	0.32	0.10	10.1	0.33	0.03
T2	5.5	0.76	0.14	12.0	0.90	0.08	7.0	0.51	0.07	11.0	0.65	0.06
T3	8.5	0.97	0.11	16.5	1.00	0.06	9.5	0.60	0.06	16.5	0.75	0.05
Fpr	*	*	*	*		*	*	*	*	*	*	*
LSD (5 %)	0.1	0.17	0.01	0.2	0.21	0.01	2.5	0.07	0.01	0.4	0.10	0.01
CV%	10.8	5.3	1.8	1.0	3.8	9.6	24.6	7.1	3.2	1.7	5.2	4.1

Trts—treatments, DM—dry matter, T1—0 Mg ha^−1^ manure (control), T2—15 Mg ha^−1^ manure, T3—30 Mg ha^−1^ manure

* p < 0.05

## Discussion

### Effect of seasonal split and single manure application on soil mineral N and N_2_O emission

Wetland smallholder farmers in subtropical Africa commonly apply manure in small doses in each vegetable cropping event while others apply large doses once in 3–4 cropping occasions. Results in this study have clearly demonstrated that small dose manure application per crop can only reduce losses of N by gaseous emissions of N_2_O at least up to the second cropping event. This trend in the N_2_O flux responses to the treatments clearly suggested that the single applications of 15 and 30 Mg manure ha^−1^ provided substantially higher masses of organic substrate (Markewich et al. 2010) for the microbial degradation processes in aerated macro-pores of the soil profile during the first two vegetable cropping events. In studies related to N transformations in soils Markewich et al. (2010) reported significant accumulations of mineralized N (NH_4_-N and NO_3_-N) in microbially driven organic matter decomposition. Upon flooding of the macro-pores with soil water in the wetland (Johnson et al. 2005; Berdad-Haughn et al. 2006) some of the NO_3_-N is subjected to denitrification (Ma et al. 2007) associated with emissions of N_2_O in soil. In addition to that, the application of cattle manure to wetland soil enhances its potential for emissions of N_2_O gas by stimulating increased microbial respiration, causing rapid oxygen consumption and consequently an increase of anaerobic conditions for the onset of denitrification processes (Yates et al. 2006; Jassal et al. 2011). Wetland soils have aerobic and anaerobic sites that allow nitrification and denitrification to take place simultaneously (Johnson et al. 2005). Since the first process produces the substrate for the second, N losses through emissions of N_2_O can be high when the two processes are associated (Snyder et al. 2009). Evidently, the application of cattle manure to wetland cropping systems has the potential of increasing the contribution of agriculture to atmospheric N_2_O loading (Markewich et al. 2010; Kamaa et al. 2011).

The mean differences in the rates of N_2_O emissions between plots amended with single basal applications and those that received split applications became progressively smaller and insignificant (p > 0.05) towards the last test crop. The decline in the vegetable plots that received single basal manure applications in evolving elevated amounts of N_2_O over those that received seasonal split manure amendments is attributed to the rapid decrease in the capacity of the plots that received single manure amendments to supply NO_3_-N, which is a substrate for the microbes that participate in the denitrification process. This decline in the content of mineralised N is a consequent of successive N uptake without replenishments, immobilization in ligno-protein complexes of humus formation (Yates et al. 2006), N_2_O gas evolving denitrification (Kamaa et al. 2011; Lesschen et al. 2011), and migration of NO_3_-N to ground water resources (Mapanda et al. 2012).

Results from the regression analysis implied that soil mineral N concentrations (NH_4_-N and NO_3_-N) displayed significant (p < 0.05) influence on the variability found in N_2_O emission. Both processes of nitrification of NH_4_-N and denitrification of NO_3-_N are thought to contribute immensely to the emissions of N_2_O although the later has been suggested to play a bigger role in the emissions (Ma et al. 2007; Smith 2012). In this study, NH_4_-N and NO_3_-N exerted comparatively equal influence on the variability found in N_2_O emissions in surface soil (r^2^ = 0.51–0.55 vs. 0.52–0.53 for first tomato crop; r^2^ = 0.51–0.57 vs. 0.50–0.53 for first rape crop; r^2^ = 0.42–0.78 vs. 0.47–0.77 for second tomato crop; r^2^ = 0.51–0.52 vs. 0.52–0.54 for second rape crop). The first stage in the decomposition of N-containing organic materials in applied manure produces ammoniacal N which is a substrate for the second process involving nitrification. Nitrogen losses in N_2_O emissions can be very high when the two processes are associated. As much as 60–70 % of fertilizer N applied to wetland crop may be volatilized as oxides of N (Snyder et al. 2009).

Results of the study imply that the seasonal split application of cattle manure in *small doses* to wetland vegetable crops as a mitigation measure for reducing the emission of N_2_O from agricultural sources is *effective* during the first two seasonal split applications. Any further seasonal split applications of cattle manure in *smaller doses* to wetland vegetable crops cannot act as an effective crop management practice that reduces the emission of N_2_O from soil.

### Effect of single and seasonal split application of manure on plant dry matter yield and soil N uptake

The nitrogen use efficiency (NUE) by the wetland vegetable crops could conceivably limit the loss of N from applied manure through gaseous emissions of N_2_O from soil. N is often cited as a limiting factor for vegetable growth in sub-tropical Africa. However, under conditions of elevated soil N, vegetable crops exhibit luxury consumption of N, leading to elevated tissue N concentration. While this pool of plant N may have benefits for vegetable plants if light levels change, it may also increase the risk of vegetable aboveground biomass quality (De Lannoy 2001). The differences between dry matter and uptake of N by the crops subjected to single basal applications and those receiving split applications of manure was smaller at the end of the experiment. This trend in the uptake of N by the crops is attributable to the initial abundance of N-rich easily decomposable organic compounds in the manure in plots that received single applications of cattle manure in four cropping events. Available forms of N became abundant in the wetland soil upon microbial decomposition of the nitrogenous compound pools, which increased root growth for the uptake of N.

The vegetable plots amended with split applications at every cropping event had initially insufficient N due to the limited quantities of manure added to create a larger net balance of mineralized N for uptake by the poorly developed root systems of the crops. The introduction of easily degradable C-rich materials in soil may have triggered a burst of microbial growth and activity that placed a burden on the limited quantities of mineralized N thereby depleting it significantly. Despite the comparatively narrow C:N ratio in the applied manure (Table 2, C:N ratio of 16.8:1), the quantities of N which was limited by the mass of manure applied in small doses may have been insufficient to introduce relatively large net balances of mineralized N after immobilization by microbes (Markewich et al. 2010), immobilization by reactive phenols from lignin degradation (Snyder et al. 2009; Kamaa et al. 2011), emissions by denitrification (Ma et al. 2007; Vasileiadou et al. 2011) and N loss by nitrate leaching (Johnson et al. 2005; Berdad-Haughn et al. 2006). The net result was a greater uptake of N in plots that received single basal applications of 15 and 30 Mg low N manure over that in vegetable plots that were subjected to split applications of 3.75 and 7.5 Mg low N manure observed in this study.

Increased dry matter accumulations on plots subjected to higher cattle manure applications was followed by higher uptake of N from the applied fertilizers (Table 7). Consequently, plots that were amended with higher rates of manure applications effectively sequestered N that may be exposed to denitrification and the associated emissions of N_2_O. With improved accumulations of dry matter and N uptake in plots subjected to higher manure applications, the applied N in manure was effectively sequestered from the wetland soil where it may be subjected to loss through emissions of N_2_O. This implies that when agronomic practices are improved through manure applications, the loss of N in N_2_O emissions may significantly decrease.

## Conclusions

Generally, it can be concluded that the seasonal application of cattle manure in small doses as a crop management mitigation measure for reducing the emissions of N_2_O from soil is effective at least up to the second seasonal split application. Thereafter, seasonal split applications of manure in smaller doses for every cropping event cannot reduce the losses of N from wetland soil in emissions of N_2_O. The improved uptake of N by the wetland vegetable crops can limit the loss of N from applied manure through gaseous emissions of N_2_O from soil.

Generally, the proportion of applied N lost as N_2_O was higher in the rape crop than in the tomato crop. It can be concluded that rape and possibly other similar leafy vegetables production has a greater potential to emit N_2_O into the atmosphere than the production of tomatoes in wetlands when cattle manure is used as a fertilizer. The loss of N in emissions of N_2_O expressed per unit mass of harvested dry matter yield of rape and tomato crops decreases significantly with increasing manure application rates, dry matter yield and N uptake. Improved agronomic practices for increased crop productivity can be used as a mitigation factor for reducing the contribution of agriculture in the global emissions of N_2_O.

## Abbreviations

AGRITEX: agriculture, technical and extension
ANOVA: analysis of variance
CA: California
DM: dry matter
FAO: Food and Agriculture Organization
Fn: fluxes of nitrous oxide
IPCC: International Panel for Climate Change
LSD: least significant difference
Mg: milligram
NUE: nitrogen use efficiency
TX: Texas
UK: United Kingdom
USDA: United States Department of Agriculture
WI: Wisconsin
